# Nephrologists’ perceptions of competencies acquired during medical residency in Nephrology and their applicability to daily clinical practice

**DOI:** 10.1590/2175-8239-JBN-2025-0024en

**Published:** 2025-11-03

**Authors:** Mariana Batista Pereira, Patrícia Oliveira Costa Eloy, Kleyton de Andrade Bastos

**Affiliations:** 1 Universidade de São Paulo, Faculdade de Medicina, Centro de Desenvolvimento em Educação Médica, São Paulo, SP, Brazil.; 2 Hospital do Servidor Público Estadual de São Paulo, Instituto de Assistência Médica ao Servidor Público Estadual, Departamento de Nefrologia, São Paulo, SP, Brazil.; 3 Universidade Cidade de São Paulo (UNICID), São Paulo, SP, Brazil.; 4 Universidade Nove de Julho (UNINOVE), São Paulo, SP, Brazil.; 5 Universidade Federal de Sergipe, Departamento de Medicina, Aracaju, SE, Brazil.

**Keywords:** Medical Residency, Nephrology, Medical Education

## Abstract

**Introduction:**

The medical residency (MR) curriculum underwent a reformulation, and in 2021, the competency matrix for MR in nephrology was published. This study aimed to evaluate nephrologists&apos; perceptions of the competencies acquired during residency and their relevance in clinical practice.

**Method:**

This was a cross-sectional study conducted using a self-administered electronic questionnaire, which included demographic data, information on professional practice, and an assessment of both the learning and the usefulness of the skills acquired during the MR program in nephrology. Participants responded to questions on a five-point Likert scale. Only nephrologists who had graduated from programs accredited by the Brazilian Ministry of Education were included.

**Results:**

A total of 163 nephrologists from different states in Brazil were included. Most considered the clinical skills acquired to be useful for practice, except for palliative care, in which 54% felt capable, although 93.2% considered it essential. Procedures for which usefulness exceeded self-reported competence included fundoscopy, insertion of permanent hemodialysis catheters, insertion of peritoneal dialysis catheters, and ultrasonography. Furthermore, less than 40% of participants reported feeling prepared to engage in management, clinical research, and teaching activities, despite perceiving their relevance.

**Conclusion:**

The study highlights nephrologists&apos; perceptions of competencies acquired during MR and underscores the need for improvements in nephrology training, particulary in management, teaching, and research.

## Introduction

Medical residency (MR) in nephrology represents a pivotal step in the training of nephrologists, providing specialized education across various areas within the specialty. In Brazil, MR programs are regulated by the Ministry of Education (MEC) and overseen by the National Medical Residency Commission, ensuring quality standards in both teaching and clinical practice^1,2^.

In recent years, there has been growing discussion about the need to enhance the training of nephrologists, aligning it more closely with market demands and the needs of the population^2,3,4^. In response, the Brazilian Society of Nephrology (BSN) published a competency matrix for MR in nephrology in 2021, outlining expectations for graduates acrossclinical, technical, administrative, and academic domains^5^.

Upon completion of the nephrology residency, physicians should be qualified to practice as nephrologists in various settings, such as outpatient clinics, emergency departments, hospitals, dialysis centers, and intensive care units (ICUs). In addition, they should also be capable of performing key procedures in the specialty, as well as conducting diagnosis and treatment of nephrological syndromes, both in the public health system and in private healthcare services^1,5^.

Despite the increase in the prevalence of kidney diseases, interest among physicians in pursuing a career in nephrology has declined globally over the years, posing a major challenge for the specialty^6,7^. To reverse this scenario, it is essential to understand the challenges faced in the daily practice of nephrologists and how MR has prepared these professionals to meet the demands of the job market.

Formal evaluations of the effectiveness of MR in nephrology and of nephrologists’ perceptions regarding the training they received are still scarce^8,9,10^. Assessing how these professionals view the acquired skills and their application in clinical practice is essential to identify gaps and opportunities for improvement in nephrology education^11,12^.

Given this context, the present study aims to evaluate the perceptions of Brazilian nephrologists regarding the skills acquired during MR and their relevance for clinical, educational, research, and management practices. The findings may inform curricular adjustments and future training strategies helping to prepare professionals who are better equipped to fmeet the challenges of contemporary nephrology^8,10,11^.

## Methods

### Design and Data Collection

A cross-sectional study was conducted using an electronic questionnaire developed by the authors (Appendices), based on the competency matrix published by the BSN^5^.

The questionnaire comprised 22 statements addressing demographic characteristics and professional profile, the learning of different skills during nephrology training (capacity) and the perceived importance of these competencies for current nephrology practice (utility). For each statement, participants should indicate their level of agreement using a 5-point Likert scale, ranging from: (1) strongly disagree; (2) disagree; (3) neutral; (4) agree; and (5) strongly agree.

Prior to data collection, the questionnaire was presented to a few nephrologists who serve as preceptors in MR programs in the specialty, with the aim of evaluating the questions for clarity, objectivity, and relevance.

After validation, in February 2023, the questionnaire was distrubedvia WhatsApp in informal specialist groups using a non-probabilistic “snowball” sampling method. The survey remained open for responses for a period of 15 days. Brazilian nephrologists who had performed nephrology residency in the country, in programs accredited by the Ministry of Education, were invited to participate in the study.

For the analysis of the results, responses were grouped. Regarding the learning of different competencies, participants who indicated items 4 and 5 in the competency questions were considered “capable”, and those who indicated items 1 and 2 were considered “not capable”. Similarly, with respect to the perceived importance of the competencies, those indicated by participants as 4 and 5 were considered “useful” in current clinical practice, and those rated as 1 and 2 were considered “not useful”.

To determine whether perceptions of MR in nephrology had changed over time, participants were divided into two groups: those who underwent their residency before 2012 and those who completed it after 2012. The year 2012 was selected as the cutoff point to balance the number of participants in each group, ensuring better statistical comparability between them. Participant characteristics and responses were then analyzed and compared.

### Statistical Analysis

Categorical variables were described as frequencies and percentages. Continuous variables were tested for normality and analyzed using appropriate statistical tests (Shapiro-Wilk, chi-square, Mann-Whitney U). The JAMOVI software was used for the analyses.

### Ethical Aspects

The study was approved by the Research Ethics Committee of the *Instituto de Assistência Médica ao Servidor Público Estadual de São Paulo* (CAAE 65556722.9.0000.5463) and all participants signed an online Free and Informed Consent Form (Appendices).

## Results

### Participant Profile

The questionnaire was completed by 164 nephrologists, with one participant excluded for having performed nephrology residency abroad. The demographic data of the participants, compared with those from the study “2023 Medical Demographics in Brazil”^13^, are presented in Table 1.

**Table 1:** General characteristics of the study participants compared with data from the 2023 medical demographics study^13^

Variable	Present study (n = 163)	2023 Medical Demographics (n = 4940)
Female gender %	54.8	52.3
Mean age, years	41.1	48.2
Age ≤ 35 years, %	26.8	16.6
Age ≥ 55 years, %	15.9	32.1
Region of practice %		
Southeast	52.1	50.3
Northeast	33.5	20.3
South	3.6	14.9
Central-West	9.7	8.9
North	0	4.3

Abbreviation – n: number; Scheffer et al.^13^

The mean age of participants was 41.1 years (range, 28–71 years), and the average time practicing as a nephrologist was 11 years (range, 0–45 years). MR in nephrology was performed in 42 programs distributed across 11 Brazilian states, with 58.5% in the state of São Paulo.

Participants reported working as nephrologists in outpatient clinics or private practices (85.9%), hospitals (85.3%), and dialysis centers (68.7%). In these settings, nephrologists perform the following procedures: insertion of temporary catheters for hemodialysis (HD) (85%), kidney biopsy (56%), insertion of permanent catheters for HD (44%), and insertion of catheters for peritoneal dialysis (PD) (38%). In addition, 46.6% are involved in teaching and research, and 29.4% work in management.

### Clinical Competencies

**Figure 1.**
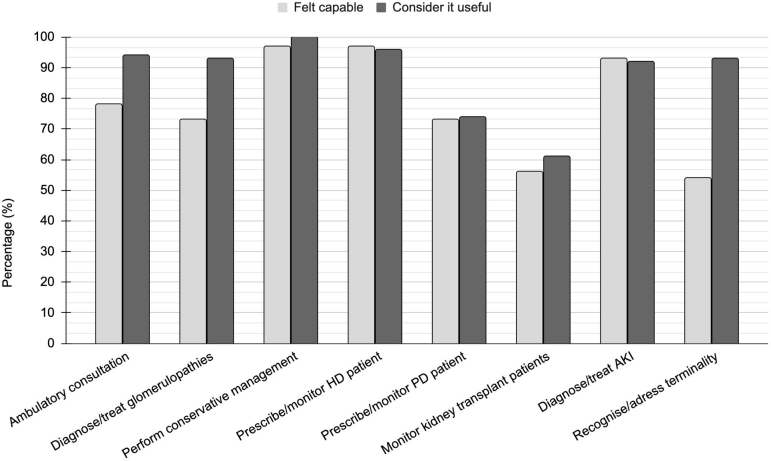
Percentage of professionals who felt capable of clinical practice after medical residency in nephrology and who recognized this skill as useful.

Figure 1 shows that most of the competencies investigated were considered useful for clinical practice by the participants and, in general, they felt capable of performing them upon completion of the MR. This perception was particularly strong for activities directly related to patient care and clinical practice, such as outpatient consultations, diagnosis and treatment of nephrological syndromes, and prescription and monitoring of renal replacement therapy (RRT). An exception was observed in palliative care training: 53.9% of participants reported feeling capable of identifying patients in the final stages of life and addressing issues related to end-of-life care after completing their MR, although 93.2% considered this knowledge useful for their current professional practice (Figure 1).

Abbreviations – HD: hemodialysis; PD: peritoneal dialysis; AKI: acute kidney injury.

**Figure 2.**
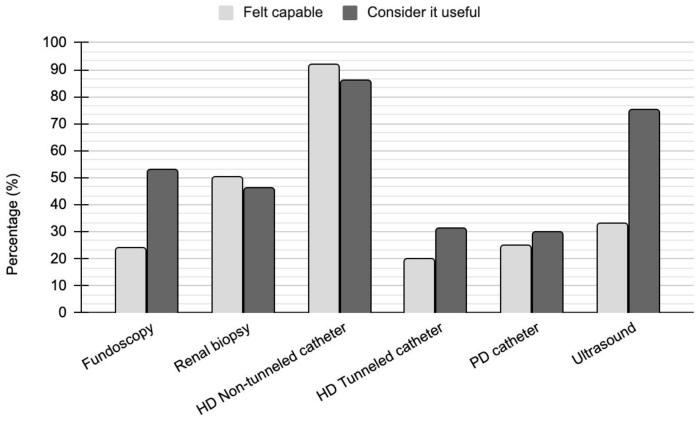
Percentage of professionals who felt capable of performing exams and procedures after medical residency in nephrology and who recognized this skill as useful.

The procedures or skills considered useful in daily practice by more than half of the participants were: performing fundus examination (FE), inserting a short-term catheter for HD, and performing ultrasound (US). However, as depicted in Figure 2, less than half of the participants felt capable of performing FE, inserting a permanent HD catheter, inserting a PD catheter, and using US as a diagnostic tool and to guide procedures. Among these, US was the skill that showed the greatest discrepancy between perceived competence (33.7%) and perceived usefulness (75.4%).

Abbreviations – HD: hemodialysis; PD: peritoneal dialysis.

The insertion of long-term catheters for HD and peritoneal catheters were the least developed skills during residency training and, correspondingly, were perceived by most respondents as less relevant to clinical practice (Figure 2).

### Management, Teaching, and Research Competencies

Most participants considered all competencies related to management, teaching and research to be useful. Nonetheless, these competencies were proportionally less recognized as having been acquired during MR in nephrology when compared to clinical competencies.

**Figure 3.**
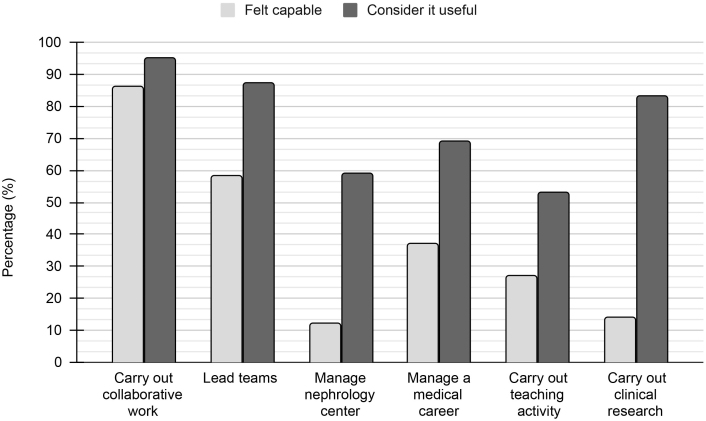
Percentage of professionals who felt capable of management, teaching and research activities after medical residency in nephrology and who recognized this skill as useful.

As shown in Figure 3, only 11.7% of participants felt capable of managing a nephrology service after completing their nephrology residency, despite 59.2% considering this skill to be useful. Regarding medical education, 26.9% reported being prepared for teaching, although 53% acknowledged its importance. Clinical research was considered essential by 84%, yet only 14% felt prepared to conduct it.

### Assessment of Competencies among Nephrologists Trained before and after 2012

The comparison between the profiles of respondents who completed MR before 2012 and after 2012 is presented in Table 2. Those who completed MR before 2012 are older (mean age 49 vs. 35 years; p < 0.001) and have higher academic qualifications, as well as greater experience in teaching, research, and management, compared to those who recently concluded MR.

**Table 2:** General and professional characteristics of the study participants according to time since completion of medical residency (mr)

Variable	Completed MR until 2012 (n = 81)	Completed MR after 2012 (n = 82)	p
Female gender, n.(%)	42 (51.9)	48 (58.5)	0.39
Mean age, years (standard deviation)	49 ± 8.0	35 ± 3.9	<0.001
MR in São Paulo – n.(%)	49 (60.4)	46 (56)	0.57
MR region – n.(%)			0.11
Southeast	64 (79.0)	61 (74.4)	
Northeast	11 (13.6)	18 (22.0)	
South	04 (4.9)	0	
Central-West	02 (2.5)	03 (3.7)	
Acts in capital – n.(%)	64 (78.0)	60 (76.9)	0.86
Region of practice %		*	0.04
Southeast	38 (47.5)	47 (57.3)	
Northeast	26 (32.5)	29 (35.4)	
South	06 (7.5)	0	
Central-West	10 (12.5)	06 (7.3)	
Educational level – n.(%)			<0.001
Medical residency	26 (32.1)	67 (81.7)	
Master&apos;s degree	25 (30.9)	13 (15.9)	
Doctorate	21 (25.9)	02 (2.4)	
Postdoctoral	09 (11.1)	0	
Field of practice – n.(%)		*	
Teaching and Research	45 (55.5)	31 (37.8)	0.02
Management	35 (43.2)	13 (15.8)	<0.001
Hospital	66 (81.4)	73 (89.0)	0.11
Outpatient Clinic or Private Practice	74 (91.3)	66 (80.4)	0.06
Dialysis Centers	53 (65.4)	59 (71.9)	0.30
Source of Updating – n.(%)			
Scientific Articles	77 (95)	75 (91.4)	0.36
Books	38 (46.9)	44 (53.6)	0.39
Courses and Congresses	63 (77.7)	61 (74.4)	0.61
Social media	25 (30.8)	42 (51.2)	0.008
Virtual classes	60 (74.0)	56 (68.3)	0.41

Abbreviation – MR: medical residency.

Note – *1 participant acts outside Brazil.

Most professionals work predominantly in care settings such as hospitals and outpatient clinics. There is a trend toward greater activity in outpatient clinics among professionals who completed their residency before 2012, compared to those who graduated after that year (91.3% vs. 80.4%; p = 0.06).

Overall, there were no significant differences between the groups regarding the main sources of professional updating, with reading scientific articles predominating, followed by participation in courses and conferences. The search for knowledge through social media was the least mentioned by respondents; however, those who completed MR after 2012 reported using it to a greater extent (51.2% vs. 30.8%; p = 0,008).

The groups also differed in their perception of certain clinical competencies acquired by the end of the training (Table 3). All participants who completed the training process after 2012 stated that they felt capable of performing conservative treatment of CKD, in contrast to 95% of those who completed the training before that period (p = 0.04). The learning perception regarding the competency “recognizing and addressing end-of-life issues” was significantly more frequent among graduates after 2012 (72% vs. 35.8%; p < 0.001). Conversely, professionals trained before 2012 reported higher percentages of self-perceived competence in performing fundoscopy (30.9% vs. 18.3%; p = 0.03) and inserting PD catheters (35.8% vs. 14.6%) (Table 3).

**Table 3:** Perception of learning (capacity) and current usefulness (utility) of competencies developed during nephrology residency, based on time since completion of training

Variable	Total (n = 163)	Completed MR until 2012 (n = 81)	Completed MR after 2012 (n = 82)	p
Carry out an outpatient consultation				
Felt capable – n.(%)	128 (78.5)	63 (77.8)	65 (79.3)	0.89
Useful – n.(%)	153 (93.8)	76 (93.8)	77 (93.9)	0.78
Diagnose and treat GN				
Felt capable – n.(%)	119 (73.0)	59 (72.8)	60 (73.2)	0.77
Useful – n.(%)	152 (93.2)	75 (92.6)	77 (93.9)	0.56
Perform conservative management				
Felt capable – n.(%)	159 (97.5)	77 (95.1)	82 (100)	0.04
Useful – n.(%)	162 (100)	81 (100)	81 (100)	
Prescribe and monitor HD patients				
Felt capable – n.(%)	158 (96.9)	77 (95.1)	81 (98.8)	0.35
Useful – n.(%)	156 (95.7)	74 (91.4)	82 (100)	0.02
Prescribe and monitor PD patients				
Felt capable – n.(%)	119 (73.0)	60 (74.1)	59 (72.0)	0.93
Useful – n.(%)	121 (74.2)	63 (77.8)	58 (70.7)	0.37
Monitor kidney transplant patients				
Felt capable – n.(%)	92 (56.4)	52 (64.2)	40 (48.8)	0.14
Useful – n.(%)	100 (61.7)	52* (65.0)	48* (59.3)	0.75
Diagnose and treat AKI				
Felt capable – n.(%)	152 (93.8)	73 (90.1)	79* (97.5)	0.13
Useful – n.(%)	150 (92.6)	71 (87.7)	79* (97.5)	0.05
Recognize and address terminality				
Felt capable – n.(%)	88 (53.9)	29 (35.8)	59 (72.0)	<0.001
Useful – n.(%)	152 (93.2)	74 (91.4)	78 (95.1)	0.62
Perform fundoscopy				
Felt capable – n.(%)	40 (24.5)	25 (30.9)	15 (18.3)	0.02
Useful – n.(%)	86 (53.1)	44* (55.0)	42 (51.2)	0.78
Perform renal biopsy				
Felt capable – n.(%)	82 (50.3)	47 (58.0)	35 (42.7)	0.14
Useful – n.(%)	75 (46.0)	41 (50.6)	34 (41.5)	0.41
Insert non-tunneled catheter for HD				
Felt capable – n.(%)	150 (92.6)	73 (90.1)	77* (95.1)	0.36
Useful – n.(%)	140 (85.9)	70 (86.4)	70 (85.4)	0.79
Insert tunneled catheter for HD				
Felt capable – n.(%)	33 (20.2)	73 (90.1)	77* (95.1)	0.59
Useful – n.(%)	51 (31.2)	70 (86.4)	70 (85.4)	0.68
Insert PD catheter				
Felt capable – n.(%)	41 (25.1)	16 (19.8)	17 (20.7)	<0.001
Useful – n.(%)	50 (30.6)	24 (29.6)	27 (32.9)	0.23
Perform ultrasound				
Felt capable – n.(%)	55 (33.7)	29 (35.8)	12 (14.6)	<0.001
Useful – n.(%)	123 (75.4)	29 (35.8)	21 (25.6)	0.001
Carry out collaborative work				
Felt capable – n.(%)	141 (86.5)	03 (3.7)	52 (63.4)	0.09
Useful – n.(%)	156 (95.7)	51 (63.0)	72 (87.8)	0.43
Lead teams				
Felt capable – n.(%)	95 (58.2)	66 (81.5)	75 (91.5)	0.22
Useful – n.(%)	142 (87.1)	78 (96.3)	78 (95.1)	0.80
Manage nephrology center				
Felt capable – n.(%)	19 (11.6)	42 (51.9)	53 (64.6)	0.14
Useful – n.(%)	96 (59.2)	71 (87.7)	71 (86.6)	0.10
Manage a medical career				
Felt capable – n.(%)	61 (37.4)	06 (7.4)	13 (15.9)	0.22
Useful – n.(%)	112 (69.1)	54* (67.5)	42 (51.2)	0.15
Carry out teaching activity				
Felt capable – n.(%)	44 (26.9)	25 (30.9)	36 (43.9)	0.05
Useful – n.(%)	87 (53.3)	57* (71.3)	55 (67.1)	0.008
Carry out clinical research				
Felt capable – n.(%)	23 (14.1)	08 (9.9)	15 (18.3)	0.20
Useful – n.(%)	136 (83.9)	65* (81.3)	71 (86.6)	0.50

Abbreviations – GN: glomerulopathies; MR: medical residency; HD: hemodialysis; PD: peritoneal dialysis; AKI: acute kidney injury.

Note – *1 participant didn&apos;t answer.

Another difference observed between the groups refers to the perceived usefulness of certain training components. Those who completed MR before 2012 attributed less usefulness to monitoring HD patients (91.4% vs. 100%; p = 0.025) and performing US (63% vs. 87.8%; p = 0.001), while considering participation in teaching activities more useful (65.4% vs. 41.5%; p = 0.008), compared to those who completed residency after 2012.

## Discussion

The present study, which included 3.32% of Brazilian nephrologists, provides an analysis of these professionals’ perceptions regarding MR in nephrology. The sample consisted of younger professionals, compared to those described in the study “2023 Medical Demographics in Brazil”^13^, which is likely explained by the use of an online data collection method and the profile of the researchers, who have greater contact with nephrologists with fewer years of practice.

The sample included professionals from MR programs across different regions in Brazil. Most participants completed their residency in the state of São Paulo, which has the highest number of MR programs^1^. This was a convenience sample, with proportionally lower representation from the South and North regions, which hinders the extrapolation of data to those locations.

The results show that, although MR adequately prepares nephrologists for essential clinical activities, there are still significant gaps in specific skills, particularly in approaching end-of-life care and in the interventional field.

Palliative care is a topic that has historically been neglected by medical education and, consequently, by MR programs in nephrology. The contrast between the lack of training and the perceived need for its inclusion in residency curricula has also been reported in several international studies^11,14,15,16^. In Brazil, palliative care was only introduced as a philosophy of care in 1980, and it was not until 2023 that its inclusion was made official in the national curricular guidelines for medical schools^17^. To date, many nephrology services, including some that offer residency programs, still lack well-established guidelines on the subject^18^.

Our fidings suggest that palliative care training has improved over the last decade and that its inclusion in the BSN competency matrix^5^ has increased awareness of the issue among MR programs. However, greater emphasis on the topic is needed through its inclusion in educational projects and theoretical course syllabi, as well as through specific practical training, including the possibility of interdisciplinary experiences in specialized palliative care services.

The learning of the other clinical competencies analyzed was similar among participants who completed MR before and after 2012, except for the perception regarding conservative treatment of CKD, which was higher among those who completed residency more recently.

Regarding the usefulness of different clinical competencies, participants who had completed their residency earlier reported lower perceived usefulness of the competency “prescribing and monitoring HD” compared to more recent graduates. This finding may be related to differences in the professional practice profile between the groups: nephrologists who graduated before 2012 reported greater involvement in outpatient clinics or private practices, while those who graduated after 2012 reported more frequent work in hospitals and HD centers.

Among the evaluated procedures, fundoscopy, insertion of a permanent catheter for HD, insertion of a peritoneal catheter, and the use of bedside ultrasound were those in which nephrologists considered themselves least capable of performing upon completion of the MR.

According to some authors, FE should become an integral part of the general physical examination^19,20^. However, its teaching since medical school and its regular practice by non-ophthalmologists are neglected, possibly due to the technical difficulties these professionals face when performing and interpreting the exam^19,20^. This skill has likely been historically less emphasized in MR, as it has fallen out of routine nephrology practice over the years.

Recent debate has focused on whether procedures previously considered essential to nephrology practice, such as HD catheter insertion and kidney biopsy, should continue to be performed by nephrologists themselves^21,22,23,24,24^. In the present study, it was observed that, in most of the centers evaluated, nephrologists perform both procedures. However, only HD temporary catheter insertion was considered useful by most participants (only 46% consider performing kidney biopsy a useful skill in current practice).

Several factors may contribute to these findings, including excessive workload, inadequate remuneration, logistical challenges, lack of opportunities for practice, and absence of specific training^23,24^. The learning process follows the logic of the service in which the resident is placed, and many of these settings do not even offer specific training opportunities, since different procedures are performed by professionals from other specialties, such as surgeons and radiologists^24^.

Regarding peritoneal catheter insertion, the scenario proves even more challenging: only 14.2% of respondents who graduated after 2012 reported having developed this competency during MR, less than half of the percentage observed in the other group (35.8%). This may be partly explained by the progressive reduction in the use of PD as a therapeutic modality in Brazil^25^, considering that some MR services do not even offer this modality.

These results raise significant questions about the procedures performed by nephrologists. As previously evidenced, the involvement of different professionals has been demonstrated to result in the fragmentation of patient care, delays in treatment initiation, and increased healthcare costs^26,27^. Furthermore, a previous study of 239 practicing nephrologists in Brazil found that 90% of respondents expressed interest in receiving further training in interventional nephrology^28^. However, there are no regulations regarding the minimum number of procedures required for certification of nephrologists in these techniques. Additionally, the availability of a specialized service with physicians from other areas, such as vascular medicine, general surgery, and urology, is necessary to manage possible complications^23,24,29^.

Given this scenario, it is essential to reassess training practices. If these procedures continue to be considered mandatory skills during nephrology MR, programs must implement effective teaching and learning strategies. An alternative would be to offer optional internships at specialized centers, aimed at residents with a specific interest in interventional nephrology, who could then serve as multipliers within their respective workplaces.

The use of US has been recommended to guide HD catheter insertion, to perform kidney biopsies and, more recently, with the introduction of point-of-care US (POCUS), as a diagnostic tool^5,30^. There are reports indicating that the use of US improves patient safety, increases satisfaction with care, and reduces treatment costs^11,30^. However, for its effective implementation, it is essential to have access to US equipment and preceptors qualified to teach this practice^31^. This study shows that bedside US training is considered useful by nephrologists, with an increasing number of professionals undergoing training, particularly those who have recently completed MR training.

The scenario described in this study reflects an internationally observed trend. Similarly, a survey conducted with nephrology residents in Italy highlighted the need to improve training in US, palliative care, clinical nutrition, and vascular access^32^.

In addition to patient care, MR should enable nephrologists to expand their curriculum and develop competencies in management, leadership, research, and teaching. In this study, most nephrologists considered that MR did not provide them with the development of these specific competencies, despite considering them useful for their current practice. International studies support these findings. A survey conducted in New Zealand demonstrated adequate preparation in most clinical areas but revealed weaknesses in the areas of research and management^12^. A study conducted by Berns in 2010 revealed that 70% of participants reported receiving little or no training in nephrology service management^10^.

Acquiring a general understanding of management is essential for nephrologists, who generally work in interdisciplinary settings, often leading teams, whether or not they hold administrative positions^4,5,11^. To develop their learning in this area, residents should receive formal training in leadership and management. This could include participation in practical administrative activities, such as management meetings, team planning, and organizing workflow processes, in addition to theoretical-practical modules on topics such as time management, nonviolent communication, feedback, people management, financial management, administration, billing, contracts, and healthcare regulations.

The results also highlight the poor preparation during residency for teaching and research activities, which may negatively impact the training of future nephrologists. Increasing the participation of nephrologists in academic activities is a potential strategy to reverse the declining interest in the specialty^7,33^. To this end, it is essential that residents receive training in the principles of adult learning, educational planning, teaching strategies, and assessment, and are actively involved in academic activities such as lectures, seminars, and supervised preceptorship. Furthermore, they should be encouraged to participate in research projects developed within their institutions and have access to structured training in scientific methodology, addressing topics ranging from study design to critical analysis of the literature, scientific writing, and academic output.

The quality of nephrologist training is intrinsically linked to the existence of MR programs with structured educational planning that are comprehensive, up-to-date, and responsive to developments in medical practice. Systematic and continuous evaluation of these programs is essential to ensure that training keeps pace with the demands of the society, the healthcare system, and the specialty itself^12^.

### Limitations

The present study has limitations. The questionnaire was developed by the authors, its content was reviewed and refined based on suggestions from some nephrologists; however, it was not validated prior to its application. Although it was based on the competency matrix of the BSN, the instrument did not cover all the competencies outlined in the document.

In addition, the questionnaire was distributed through the authors’ personal contacts, which may have led to the selection of a younger and more digitally connected population. Despite the efforts made, the survey involved a limited number of nephrologists, which may have introduced selection bias. Most participants were from the Southeast region, with low representation from the North and South regions of Brazil, which may limit the generalizability of the results to other regions of the country.

The results rely on participants’ subjective perceptions of the acquisition and usefulness of competencies during MR, which may be influed by recall bias. No objective assessments were conducted to measure the actual mastery of these competencies or their applicability in the healthcare system or the community.

Finally, the study included nephrologists who completed their training in different time periods. As residency programs have evolved over time, discrepancies in learning perceptions among different generations of specialists may exist.

### Clinical Implications

The findings of this study highlight the need for continuous improvement of MR in nephrology, with an emphasis on enhancing education in palliative care, bedside US, healthcare management, and medical education. The teaching-learning process during MR for procedures such as kidney biopsy, insertion of permanent HD catheters, and PD catheters should be reevaluated.

The implementation of systematic evaluations of MR program graduates may provide valuable input for more effective curricular adjustments, aligning medical training with the demands of society and the healthcare system. In addition, these assessments could guide the development of continuing education programs, ensuring that nephrologists are prepared for the contemporary challenges of the specialty, thereby promoting more efficient care.

## Conclusion

MR in nephrology in Brazil fulfills its educational role; however, important gaps remain in areas such as palliative care, interventional procedures, teaching, research, and management. These gaps need to be addressed to ensure more comprehensive training for nephrologists.

This study may contribute to improving nephrology MR programs through systematic evaluation of graduates and the implementation of continuing education programs, enabling curricular adjustments that adress the needs of both society and the specialty.

## Data Availability

The datasets generated and/or analyzed during the present study are available upon reasonable request to the corresponding author.

## Supplementary Material

The following online material is available for this article:

Anexo 1 Questionário.

Anexo 2 Termo de Consentimento Livre e Esclarecido.
