# Supplementary material for: Nephrologists’ perceptions of competencies acquired during medical residency in Nephrology and their applicability to daily clinical practice
Source: J Bras Nefrol. 2025 Nov 3;47(4):e20250024. doi: 10.1590/2175-8239-JBN-2025-0024en (PMC12604310; doi:10.1590/2175-8239-JBN-2025-0024en)
Supplement: Anexo 1 [file 2175-8239-jbn-47-4-e20250024-suppl1.pdf]

Braz. J. Nephrol.
https://doi.org/10.1590/2175-8239-JBN-2025-0024pt

Material Suplementar para "Percepção dos nefrologistas sobre as competências adquiridas durante a residência médica em Nefrologia e sua aplicabilidade na prática clínica diária"

Anexos

Anexo 1 - Questionário

Caracterização do participante e do serviço que atua

Realizou residência médica em nefrologia?
Gênero:
Idade:
Onde realizou a residência médica? (hospital/programa)
Quando terminou a residência médica? (mês/ano)
Cidade e estado de atuação:
Grau de instrução:

- Pós graduação/ Residência médica
- Mestrado
- Doutorado
- Pós-doutorado

Como nefrologista, atua em qual área:

- Ensino/ Pesquisa
- Gestão
- Hospital
- Ambulatório / consultório
- Clínica de diálise

Nos serviços onde você trabalha, quem implanta/faz:

- Cateter peritoneal
- Cateter tunelizado
- Cateter não tunelizado
- Biópsia renal

Como você se atualiza e busca os conhecimentos que não foram adquiridos na residência?

- Artigos científicos
- Livros
- Cursos ou congressos
- Redes sociais
- Aulas virtuais

Questões:

Para responder as perguntas a seguir, utilize a escala de 0 a 5 pontos, onde:

1: discordo totalmente

2: discordo

3: não tenho certeza

4: concordo

5: concordo totalmente

<table>
<tr><td colspan="2">1. Realizar uma consulta ambulatorial de nefrologia</td></tr>
<tr><td>1.1 Após o término da residência em nefrologia, me sentia apto(a)
<table><tr><td>1</td><td>2</td><td>3</td><td>4</td><td>5</td></tr></table>
discordo totalmente discordo neutro concordo concordo totalmente</td><td>1.2 Este conhecimento é útil para a minha vivência atual
<table><tr><td>1</td><td>2</td><td>3</td><td>4</td><td>5</td></tr></table>
discordo totalmente discordo neutro concordo concordo totalmente</td></tr>
<tr><td colspan="2">2. Realizar e interpretar um exame de fundo de olho</td></tr>
<tr><td>2.1 Após o término da residência em nefrologia, me sentia apto(a)
<table><tr><td>1</td><td>2</td><td>3</td><td>4</td><td>5</td></tr></table>
discordo totalmente discordo neutro concordo concordo totalmente</td><td>2.2 Este conhecimento é útil para a minha vivência atual
<table><tr><td>1</td><td>2</td><td>3</td><td>4</td><td>5</td></tr></table>
discordo totalmente discordo neutro concordo concordo totalmente</td></tr>
<tr><td colspan="2">3. Diagnosticar e tratar uma glomerulopatia</td></tr>
</table>

3.1 Após o término da residência em nefrologia, me sentia apto(a)	3.2 Este conhecimento é útil para a minha vivência atual
1 | 2 | 3 | 4 | 5	1 | 2 | 3 | 4 | 5
discordo totalmente · discordo · neutro · concordo · concordo totalmente	discordo totalmente · discordo · neutro · concordo · concordo totalmente

4. Realizar biópsia renal

4.1 Após o término da residência em nefrologia, me sentia apto(a)	4.2 Este conhecimento é útil para a minha vivência atual
1 | 2 | 3 | 4 | 5	1 | 2 | 3 | 4 | 5
discordo totalmente · discordo · neutro · concordo · concordo totalmente	discordo totalmente · discordo · neutro · concordo · concordo totalmente

5. Realizar tratamento conservador de doença renal crônica e indicar terapia substitutiva renal

5.1 Após o término da residência em nefrologia, me sentia apto(a)	5.2 Este conhecimento é útil para a minha vivência atual
1 | 2 | 3 | 4 | 5	1 | 2 | 3 | 4 | 5
discordo totalmente · discordo · neutro · concordo · concordo totalmente	discordo totalmente · discordo · neutro · concordo · concordo totalmente

6. Realizar implante de acesso vascular temporário para hemodiálise

6.1 Após o término da residência em nefrologia, me sentia apto(a)	6.2 Este conhecimento é útil para a minha vivência atual
1 | 2 | 3 | 4 | 5	1 | 2 | 3 | 4 | 5

<table>
<tr><td>discordo totalmente discordo neutro concordo concordo totalmente</td><td>discordo totalmente discordo neutro concordo concordo totalmente</td></tr>
<tr><td colspan="2">7. Realizar implante de cateter tunelizado para hemodiálise</td></tr>
<tr><td>7.1 Após o término da residência em nefrologia, me sentia apto(a)<table><tr><td>1</td><td>2</td><td>3</td><td>4</td><td>5</td></tr></table>discordo totalmente discordo neutro concordo concordo totalmente</td><td>7.2 Este conhecimento é útil para a minha vivência atual<table><tr><td>1</td><td>2</td><td>3</td><td>4</td><td>5</td></tr></table>discordo totalmente discordo neutro concordo concordo totalmente</td></tr>
<tr><td colspan="2">8. Prescrever e acompanhar pacientes em hemodiálise</td></tr>
<tr><td>8.1 Após o término da residência em nefrologia, me sentia apto(a)<table><tr><td>1</td><td>2</td><td>3</td><td>4</td><td>5</td></tr></table>discordo totalmente discordo neutro concordo concordo totalmente</td><td>8.2 Este conhecimento é útil para a minha vivência atual<table><tr><td>1</td><td>2</td><td>3</td><td>4</td><td>5</td></tr></table>discordo totalmente discordo neutro concordo concordo totalmente</td></tr>
<tr><td colspan="2">9. Realizar implante de cateter para diálise peritoneal</td></tr>
<tr><td>9.1 Após o término da residência em nefrologia, me sentia apto(a)<table><tr><td>1</td><td>2</td><td>3</td><td>4</td><td>5</td></tr></table>discordo totalmente discordo neutro concordo concordo totalmente</td><td>9.2 Este conhecimento é útil para a minha vivência atual<table><tr><td>1</td><td>2</td><td>3</td><td>4</td><td>5</td></tr></table>discordo totalmente discordo neutro concordo concordo totalmente</td></tr>
<tr><td colspan="2">10. Prescrever e acompanhar pacientes em diálise peritoneal</td></tr>
<tr><td>10.1 Após o término da residência em nefrologia, me sentia apto(a)</td><td>10.2 Este conhecimento é útil para a minha vivência atual</td></tr>
</table>

<table>
<tr><td><table><tr><td>1</td><td>2</td><td>3</td><td>4</td><td>5</td></tr></table>discordo totalmente discordo neutro concordo concordo totalmente</td><td><table><tr><td>1</td><td>2</td><td>3</td><td>4</td><td>5</td></tr></table>discordo totalmente discordo neutro concordo concordo totalmente</td></tr>
<tr><td colspan="2">11. Realizar o acompanhamento de um paciente com transplante renal</td></tr>
<tr><td>11.1 Após o término da residência em nefrologia, me sentia apto(a)<table><tr><td>1</td><td>2</td><td>3</td><td>4</td><td>5</td></tr></table>discordo totalmente discordo neutro concordo concordo totalmente</td><td>11.2 Este conhecimento é útil para a minha vivência atual<table><tr><td>1</td><td>2</td><td>3</td><td>4</td><td>5</td></tr></table>discordo totalmente discordo neutro concordo concordo totalmente</td></tr>
<tr><td colspan="2">12. Diagnosticar, tratar e prescrever terapia de suporte renal para um paciente com injúria renal aguda</td></tr>
<tr><td>12.1 Após o término da residência em nefrologia, me sentia apto(a)<table><tr><td>1</td><td>2</td><td>3</td><td>4</td><td>5</td></tr></table>discordo totalmente discordo neutro concordo concordo totalmente</td><td>12.2 Este conhecimento é útil para a minha vivência atual<table><tr><td>1</td><td>2</td><td>3</td><td>4</td><td>5</td></tr></table>discordo totalmente discordo neutro concordo concordo totalmente</td></tr>
<tr><td colspan="2">13. Realizar ultrassonografia à beira do leito como ferramenta diagnóstica e para punção venosa</td></tr>
<tr><td>13.1 Após o término da residência em nefrologia, me sentia apto(a)<table><tr><td>1</td><td>2</td><td>3</td><td>4</td><td>5</td></tr></table>discordo discordo neutro concordo concordo</td><td>13.2 Este conhecimento é útil para a minha vivência atual<table><tr><td>1</td><td>2</td><td>3</td><td>4</td><td>5</td></tr></table>discordo discordo neutro concordo concordo</td></tr>
</table>

totalmente totalmente	totalmente totalmente
14. Reconhecer pacientes com doenças renais em fase final de vida e conversar com estes e seus familiares sobre a terminalidade	
14.1 Após o término da residência em nefrologia, me sentia apto(a)<br>1 | 2 | 3 | 4 | 5<br>discordo totalmente, discordo, neutro, concordo, concordo totalmente	14.2 Este conhecimento é útil para a minha vivência atual<br>1 | 2 | 3 | 4 | 5<br>discordo totalmente, discordo, neutro, concordo, concordo totalmente
15. Exercer o trabalho colaborativo interprofissional	
15.1 Após o término da residência em nefrologia, me sentia apto(a)<br>1 | 2 | 3 | 4 | 5<br>discordo totalmente, discordo, neutro, concordo, concordo totalmente	15.2 Este conhecimento é útil para a minha vivência atual<br>1 | 2 | 3 | 4 | 5<br>discordo totalmente, discordo, neutro, concordo, concordo totalmente
16. Liderar uma equipe de assistência ao paciente com doença renal	
16.1 Após o término da residência em nefrologia, me sentia apto(a)<br>1 | 2 | 3 | 4 | 5<br>discordo totalmente, discordo, neutro, concordo, concordo totalmente	16.2 Este conhecimento é útil para a minha vivência atual<br>1 | 2 | 3 | 4 | 5<br>discordo totalmente, discordo, neutro, concordo, concordo totalmente
17. Gerenciar serviços de nefrologia	
17.1 Após o término da residência em nefrologia, me sentia apto(a)	17.2 Este conhecimento é útil para a minha vivência atual

<table>
<tr>
<td><table><tr><td>1</td><td>2</td><td>3</td><td>4</td><td>5</td></tr></table>
discordo totalmente discordo neutro concordo concordo totalmente</td>
<td><table><tr><td>1</td><td>2</td><td>3</td><td>4</td><td>5</td></tr></table>
discordo totalmente discordo neutro concordo concordo totalmente</td>
</tr>
<tr><td colspan="2">18. Realizar atividades docentes</td></tr>
<tr>
<td>18.1 Após o término da residência em nefrologia, me sentia apto(a)
<table><tr><td>1</td><td>2</td><td>3</td><td>4</td><td>5</td></tr></table>
discordo totalmente discordo neutro concordo concordo totalmente</td>
<td>18.2 Este conhecimento é útil para a minha vivência atual
<table><tr><td>1</td><td>2</td><td>3</td><td>4</td><td>5</td></tr></table>
discordo totalmente discordo neutro concordo concordo totalmente</td>
</tr>
<tr><td colspan="2">19. Realizar uma pesquisa clínica em nefrologia</td></tr>
<tr>
<td>19.1 Após o término da residência em nefrologia, me sentia apto(a)
<table><tr><td>1</td><td>2</td><td>3</td><td>4</td><td>5</td></tr></table>
discordo totalmente discordo neutro concordo concordo totalmente</td>
<td>19.2 Este conhecimento é útil para a minha vivência atual
<table><tr><td>1</td><td>2</td><td>3</td><td>4</td><td>5</td></tr></table>
discordo totalmente discordo neutro concordo concordo totalmente</td>
</tr>
<tr><td colspan="2">20. Realizar a gestão da sua carreira</td></tr>
<tr>
<td>20.1 Após o término da residência em nefrologia, me sentia apto(a)
<table><tr><td>1</td><td>2</td><td>3</td><td>4</td><td>5</td></tr></table>
discordo totalmente discordo neutro concordo concordo totalmente</td>
<td>20.2 Este conhecimento é útil para a minha vivência atual
<table><tr><td>1</td><td>2</td><td>3</td><td>4</td><td>5</td></tr></table>
discordo totalmente discordo neutro concordo concordo totalmente</td>
</tr>
</table>

21. O que você acha que faltou de aprendizado durante sua residência médica? Se pudesse dar sugestões para melhoria do seu programa de residência, quais seriam?
