# Supplementary material for: Nephrologists’ perceptions of competencies acquired during medical residency in Nephrology and their applicability to daily clinical practice
Source: J Bras Nefrol. 2025 Nov 3;47(4):e20250024. doi: 10.1590/2175-8239-JBN-2025-0024en (PMC12604310; doi:10.1590/2175-8239-JBN-2025-0024en)
Supplement: Anexo 2 [file 2175-8239-jbn-47-4-e20250024-suppl2.pdf]

*Braz. J. Nephrol.*
https://doi.org/10.1590/2175-8239-JBN-2025-0024pt

# Material Suplementar para “Percepção dos nefrologistas sobre as competências adquiridas durante a residência médica em Nefrologia e sua aplicabilidade na prática clínica diária”

**Anexo 2 - Termo de Consentimento Livre e Esclarecido**

Você está sendo convidado(a) a participar como voluntário da pesquisa intitulada “RESIDÊNCIA MÉDICA SOB A ÓTICA DO NEFROLOGISTA”, realizada pelo centro de desenvolvimento e educação médica da Faculdade de Medicina da Universidade de São Paulo (CEDEM FMUSP) e pelo Instituto de Assistência Médica ao Servidor Público de Estado de São Paulo (IAMSPE). O objetivo principal da pesquisa é avaliar a percepção dos médicos nefrologistas sobre as competências adquiridas durante a residência médica e a importância destas para sua prática diária em Nefrologia, nas áreas clínicas, de educação, pesquisa e gestão. Para isso, será aplicado questionário virtual do Google Forms, com perguntas objetivas e subjetivas, aos egressos de programas de residência de nefrologia do Brasil. O tempo estimado para preenchimento do questionário é de 10 minutos.

O resultado dos dados coletados será divulgado como artigo científico publicado em revista e como trabalho de conclusão do curso do CEDEM FMUSP, e tem como benefício permitir maior conhecimento do processo de aprendizagem dos programas de residência médica em nefrologia.

Como garantia da privacidade e confidencialidade dos dados, não será solicitado identificação pessoal e apenas os pesquisadores terão acesso às informações obtidas. Em qualquer momento o participante pode se retirar da pesquisa sem qualquer tipo de prejuízo a sua pessoa.

O risco do estudo é de constrangimento, já que o(a) participante pode se sentir desconfortável com itens do questionário. Caso isso aconteça, ele poderá retirar seu consentimento do estudo a qualquer momento ou não responder à pergunta, sem qualquer tipo de prejuízo a sua pessoa.

A pesquisa beneficiará os participantes, pois permitirá uma avaliação crítica sobre a residência médica e a sua prática clínica atual, ajudando a repensar a formação de novos nefrologistas.

Esta pesquisa foi elaborada de acordo com as diretrizes e normas regulamentadoras de pesquisa envolvendo seres humanos e atende às resoluções 466/2012 e 510/2016, do Conselho Nacional de Saúde do Ministério de Saúde – Brasília – DF. Os pesquisadores asseguram a garantia de indenização e assistência integral mediante eventuais danos decorrentes da pesquisa (Resolução 466/2012/CONEP).

Este documento e a pesquisa foram revisados e aprovados pelo Comitê de Ética em Pesquisa com Seres Humanos do Instituto de Assistência Médica ao Servidor Público de Estado de São Paulo (IAMSPE). Além disso, por ser documento de permissão virtual, segue as recomendações da Carta Circular N° 1/2021/CONEP/SECNS/MS.

Para informações, dúvidas e solicitações, entrar em contato com os pesquisadores:

Kleyton de Andrade Bastos, email: kleytonbastos@yahoo.com.br

Mariana Batista Pereira, email: marianabpereira78@gmail.com

Patrícia Oliveira Costa, email: patriciacosta.med@gmail.com

Se houver alguma dúvida quanto à parte ética do projeto, por favor entre em contato com o Comitê de Ética em Pesquisa do IAMSPE, situado na Av. Ibirapuera, 981, 1º andar, Sala 106 - pelo telefone (11) 4573.8175 ou e-mail: cepiamspe@iamspe.sp.gov.br (horário: das 7h às 13h).

Ao final do questionário, caso você deseje, pode optar por receber uma cópia do TCLE e das suas respostas pelo email fornecido. A impressão do TCLE serve como comprovante do mesmo.

**Declaração de consentimento**

Declaro que fui informado (a) e esclarecido (a) sobre o presente documento, entendendo todos os termos acima expostos, e que estou ciente e de acordo em participar do estudo “Residência em Nefrologia sob a ótica do nefrologista”.

Local e data:

| ______________________________ | ______________________________ |
|---|---|
| Nome do participante | Mariana Batista Pereira |
| Assinatura do Participante | Assinatura do pesquisador |
