# Supplementary material for: Nephrologists’ perceptions of competencies acquired during medical residency in Nephrology and their applicability to daily clinical practice
Source: J Bras Nefrol. 2025 Nov 3;47(4):e20250024. doi: 10.1590/2175-8239-JBN-2025-0024en (PMC12604310; doi:10.1590/2175-8239-JBN-2025-0024en)
Supplement: Translation

# Translation: Percepção dos nefrologistas sobre as competências adquiridas durante a residência médica em Nefrologia e sua aplicabilidade na prática clínica diária

**DOI:** 10.1590/2175-8239-JBN-2025-0024pt

**Authors:** Mariana Batista Pereira, Patrícia Oliveira Costa Eloy, Kleyton de Andrade Bastos

**Affiliations:** 1 Universidade de São Paulo, Faculdade de Medicina, Centro de Desenvolvimento em Educação Médica, São Paulo, SP, Brasil.; 2 Hospital do Servidor Público Estadual de São Paulo, Instituto de Assistência Médica ao Servidor Público Estadual, Departamento de Nefrologia, São Paulo, SP, Brasil.; 3 Universidade Cidade de São Paulo (UNICID), São Paulo, SP, Brasil.; 4 Universidade Nove de Julho (UNINOVE), São Paulo, SP, Brasil.; 5 Universidade Federal de Sergipe, Departamento de Medicina, Aracaju, SE, Brasil.

**Keywords:** Residência Médica, Nefrologia, Educação Médica

## Abstract

**Introdução:**

O currículo da residência médica (RM) passou por uma reformulação e, em 2021, publicou-se a matriz de competências para a RM em nefrologia. Este estudo teve como objetivo avaliar a percepção de nefrologistas sobre as competências adquiridas durante a RM e sua importância na prática clínica atual.

**Método:**

Estudo transversal, conduzido por meio de questionário eletrônico autoaplicável, que incluiu dados demográficos, informações sobre a atuação profissional e avaliação do aprendizado e da utilidade das competências adquiridas durante a RM em nefrologia. Os participantes responderam às questões em escala Likert de cinco pontos. Foram incluídos apenas nefrologistas formados em programas credenciados pelo Ministério da Educação.

**Resultados:**

Foram incluídos 163 nefrologistas de diferentes estados do Brasil. A maioria considerou as competências clínicas adquiridas e úteis para a prática, com exceção do cuidado paliativo, no qual 54% sentiram-se aptos e 93,2% o consideraram essencial. Os procedimentos em que a utilidade superou o aprendizado foram: realização de exame de fundo de olho, implante de cateter definitivo para hemodiálise, cateter para diálise peritoneal e realização de ultrassonografia. Além disso, menos de 40% dos participantes relataram sentir-se preparados para atuar em gestão, pesquisa clínica e atividades docentes, apesar da percepção da sua importância.

**Conclusão:**

O estudo destaca a percepção dos nefrologistas sobre as competências adquiridas durante a RM e aponta a necessidade de aprimoramento na formação em nefrologia, especialmente nas áreas de gestão, educação e pesquisa.

## Introdução

A residência médica (RM) em nefrologia é uma etapa essencial na formação do nefrologista, proporcionando treinamento especializado em diversas áreas da especialidade. No Brasil, a RM é regulamentada pelo Ministério da Educação (MEC) e supervisionada pela Comissão Nacional de Residência Médica, garantindo padrões de qualidade no ensino e na prática clínica^1,2^.

Nos últimos anos, houve uma crescente discussão sobre a necessidade de aprimorar a formação dos nefrologistas, tornando-a mais alinhada às demandas do mercado e às necessidades da população^2–4^. Em resposta a essas questões, a Sociedade Brasileira de Nefrologia (SBN) publicou, em 2021, a matriz de competências para a RM em nefrologia, estabelecendo diretrizes sobre o que se espera do egresso, abrangendo aspectos clínicos, técnicos, administrativos e acadêmicos^5^.

Ao concluir a RM em nefrologia, o médico deve estar apto a atuar como nefrologista em diversas áreas, como ambulatórios, emergências, hospitais, centros de diálise e unidades de terapia intensiva (UTI). Além disso, deve ser capaz de realizar procedimentos essenciais da especialidade, bem como conduzir o diagnóstico e a terapêutica das síndromes nefrológicas, tanto no Sistema Único de Saúde quanto em serviços privados^1,5^.

Apesar do aumento da prevalência de doenças renais, o interesse dos médicos em seguir carreira na área da nefrologia tem diminuído globalmente ao longo dos anos, representando um importante desafio para a especialidade^6,7^. Para reverter esse cenário, é fundamental compreender os desafios enfrentados na prática diária do nefrologista e entender como a RM tem preparado esses profissionais para as demandas do mercado de trabalho.

Ainda são escassas as avaliações formais sobre a efetividade da RM em nefrologia e sobre a percepção dos próprios nefrologistas quanto à formação recebida^8–10^. Avaliar como esses profissionais enxergam as competências adquiridas e sua aplicação na prática clínica é essencial para identificar lacunas e oportunidades de melhoria no ensino da nefrologia^11,12^.

Diante desse contexto, este estudo tem como objetivo avaliar a percepção de nefrologistas brasileiros sobre as competências adquiridas durante a RM e sua relevância para a prática clínica, educacional, de pesquisa e de gestão. Os achados podem fornecer subsídios para ajustes curriculares e para o desenvolvimento de futuras estratégias de capacitação na especialidade, contribuindo para a formação de profissionais melhor preparados para os desafios da nefrologia contemporânea^8–11^.

## Métodos

### Desenho e Coleta de Dados

Foi realizado um estudo transversal, utilizando questionário eletrônico elaborado pelos autores (Anexos), com base na matriz de competências publicada pela SBN^5^.

O questionário continha 22 afirmações que versavam sobre características demográficas e perfil profissional, o aprendizado de diferentes competências durante a RM em nefrologia (aptidões) e a percepção da importância dessas competências para a prática atual como nefrologista (utilidade). Para cada afirmação, os participantes deveriam indicar seu grau de concordância por meio de uma escala Likert de 5 pontos, variando de: (1) discordo totalmente; (2) discordo; (3) neutro; (4) concordo; e (5) concordo totalmente.

Antes do início da coleta de dados, o questionário foi apresentado a alguns nefrologistas preceptores de programas de RM da especialidade, com o objetivo de avaliar as perguntas quanto à clareza, objetividade e relevância.

Após essa validação, em fevereiro de 2023, o questionário foi enviado por WhatsApp a nefrologistas, utilizando-se de grupos informais de especialistas e da estratégia de amostragem não probabilística do tipo “bola de neve”, permanecendo aberto para respostas por um período de 15 dias. Foram convidados a participar do estudo médicos nefrologistas brasileiros que haviam realizado RM em nefrologia no país, em programas credenciados pelo MEC.

Para a análise dos resultados, as respostas foram agrupadas. Em relação ao aprendizado das diferentes competências, foram considerados “aptos” os participantes que indicaram os itens 4 e 5 nas questões de aptidões, e “inaptos” aqueles que assinalaram os itens 1 e 2. Da mesma forma, no que se refere à percepção da importância das competências, foram consideradas “úteis” na prática clínica atual aquelas indicadas pelos participantes como 4 e 5, e “inúteis”, as registradas como 1 e 2.

Para verificar se houve diferença na percepção da RM em nefrologia ao longo do tempo, os participantes foram divididos em dois grupos: aqueles que realizaram RM em nefrologia antes de 2012 e os que a realizaram após 2012. O ano de 2012 foi escolhido como ponto de corte para equilibrar o número de participantes em cada grupo, assegurando melhor comparabilidade estatística entre eles. As características dos participantes e suas respostas foram então analisadas e comparadas.

### Análise Estatística

As variáveis categóricas foram descritas em frequências e porcentagens. As variáveis contínuas foram testadas quanto à normalidade e analisadas por meio de testes estatísticos adequados (Shapiro-Wilk, qui-quadrado, U de Mann-Whitney). O software JAMOVI foi utilizado para as análises.

### Aspectos Éticos

O estudo foi aprovado pelo Comitê de Ética em Pesquisa do Instituto de Assistência Médica ao Servidor Público Estadual de São Paulo (CAAE 65556722.9.0000.5463) e todos os participantes assinaram, de forma on-line, o Termo de Consentimento Livre e Esclarecido (Anexos).

## Resultados

### Perfil dos Participantes

O questionário foi respondido por 164 nefrologistas, sendo excluído um participante por ter realizado RM em nefrologia no exterior. Os dados demográficos dos participantes, comparados com os do estudo “Demografia Médica no Brasil 2023”^13^, estão apresentados na Tabela 1.

**Tabela 1:** Características gerais dos participantes do estudo em comparação com os dados do estudo “demografia médica 2023”^13^

Variável	Presente estudo (N = 163)	Demografia médica 2023 (N = 4940)
Gênero feminino, %	54,8	52,3
Idade, média, anos	41,1	48,2
Idade ≤ 35 anos, %	26,8	16,6
Idade ≥ 55 anos, %	15,9	32,1
Região de atuação, %		
Sudeste	52,1	50,3
Nordeste	33,5	20,3
Sul	3,6	14,9
Centro-Oeste	9,7	8,9
Norte	0	4,3

Abreviação – N: número. Scheffer et al.^13^

A idade média dos participantes foi de 41,1 anos (28–71 anos) e o tempo médio de atuação como nefrologista foi de 11 anos (0–45 anos). A RM em nefrologia foi realizada em 42 programas, distribuídos por 11 estados brasileiros, sendo 58,5% no estado de São Paulo.

Os participantes atuam como nefrologistas em ambulatórios ou consultórios (85,9%), hospitais (85,3%) e clínicas de diálise (68,7%). Nesses locais, os seguintes procedimentos são realizados pelos nefrologistas: implante de cateter provisório para hemodiálise (HD) (85%), biópsia renal (56%), passagem de cateter definitivo para HD (44%) e passagem de cateter de diálise peritoneal (DP) (38%). Além disso, 46,6% trabalham com ensino e pesquisa e 29,4% com gestão.

### Competências Clínicas

A Figura 1 mostra que a maioria das competências pesquisadas foi considerada útil à prática clínica pelos participantes e, de modo geral, eles se sentiam aptos a executá-las ao final da RM. Essa percepção foi especialmente evidente em relação às atividades diretamente assistenciais e vinculadas à prática clínica, como a realização de consultas ambulatoriais, o diagnóstico e tratamento de síndromes nefrológicas, além da prescrição e acompanhamento da terapia renal substitutiva (TRS). A exceção observada foi no aprendizado sobre cuidados paliativos: 53,9% dos participantes relataram sentir-se aptos a reconhecer pacientes em fase final de vida e abordar questões relacionadas à terminalidade após a RM, embora 93,2% considerassem esse conhecimento útil para a prática profissional atual (Figura 1).

**Figura 1.** Porcentagem de profissionais que se sentiam aptos para atuação clínica após a residência médica em nefrologia e que reconhece como útil esta competência.

Abreviações – GN: glomerulopatia; HD: hemodiálise; DP: diálise peritoneal; IRA: injúria renal aguda.

Os procedimentos ou habilidades consideradas úteis na prática diária por mais da metade dos participantes foram: a realização de exame de fundo de olho (FO), a passagem de cateter de curta permanência para HD e a realização de ultrassonografia (USG). No entanto, conforme demonstrado na Figura 2, menos da metade dos participantes se sentia apta a realizar FO, implantar cateter definitivo para HD, implantar cateter de DP e utilizar a USG como ferramenta diagnóstica e para guiar procedimentos. Dentre essas, a realização de USG foi a habilidade que apresentou maior discrepância entre a sensação de aptidão (33,7%) e a percepção de utilidade (75,4%).

**Figura 2.** Porcentagem de profissionais que se sentiam aptos para a realização de exames e procedimentos após a residência médica em nefrologia e que reconhece como útil esta habilidade.

Abreviações – HD: hemodiálise; DP: diálise peritoneal.

O implante de cateter de longa permanência para HD e o de cateter peritoneal foram as habilidades menos desenvolvidas durante a formação e, de forma correspondente, foram percebidas pela maioria como de menor relevância para a prática clínica (Figura 2).

### Competências em Gestão, Ensino e Pesquisa

Todas as competências abordadas relativas à gestão, ensino e pesquisa foram consideradas úteis pela maioria dos participantes, apesar de terem sido proporcionalmente menos reconhecidas como aprendidas durante a RM em nefrologia, quando comparadas às competências clínicas.

Como mostra a Figura 3, após o término da RM em nefrologia, apenas 11,7% dos participantes se sentiam aptos a gerenciar um serviço de nefrologia, apesar de 59,2% considerarem essa habilidade útil. Em relação à educação médica, 26,9% afirmaram estar preparados para a docência, embora 53% reconhecessem sua importância. A pesquisa clínica foi considerada essencial por 84%, mas apenas 14% relataram sentir-se preparados para realizá-la.

**Figura 3.** Porcentagem de profissionais que se sentiam aptos para atividades de gestão, ensino e pesquisa após a residência médica em nefrologia e que reconhece como útil esta habilidade.

### Avaliação das Competências Entre os Nefrologistas Formados Antes e Após 2012

A comparação entre o perfil dos participantes que concluíram a RM antes de 2012 e após 2012 está apresentada na Tabela 2. Aqueles que finalizaram a RM antes de 2012 são mais velhos (média de idade de 49 vs. 35 anos; p < 0,001) e apresentam maior titulação acadêmica, além de maior atuação nas áreas de ensino, pesquisa e gestão, em comparação aos que concluíram a RM mais recentemente.

**Tabela 2:** Características gerais e profissionais dos participantes do estudo divididos em função do tempo de conclusão da residência médica (rm)

Variável	Conclusão até 2012 (N = 81)	Conclusão após 2012 (N = 82)	p
Gênero feminino, N (%)	42 (51,9)	48 (58,5)	0,39
Idade, média, anos (desvio-padrão)	49 ± 8,0	35 ± 3,9	<0,001
RM em São Paulo, N (%)	49 (60,4)	46 (56)	0,57
Região da RM, N (%)			0,11
Sudeste	64 (79,0)	61 (74,4)	
Nordeste	11 (13,6)	18 (22,0)	
Sul	04 (4,9)	0	
Centro-Oeste	02 (2,5)	03 (3,7)	
Atuação em capital, N (%)	64 (78,0)	60 (76,9)	0,86
Região de atuação, N (%)		*	0,04
Sudeste	38 (47,5)	47 (57,3)	
Nordeste	26 (32,5)	29 (35,4)	
Sul	06 (7,5)	0	
Centro-Oeste	10 (12,5)	06 (7,3)	
Grau de instrução, N (%)			<0,001
Residência médica	26 (32,1)	67 (81,7)	
Mestrado	25 (30,9)	13 (15,9)	
Doutorado	21 (25,9)	02 (2,4)	
Pós-doutorado	09 (11,1)	0	
Área de atuação, N (%)		*	
Ensino e pesquisa	45 (55,5)	31 (37,8)	0,02
Gestão	35 (43,2)	13 (15,8)	<0,001
Hospital	66 (81,4)	73 (89,0)	0,11
Ambulatório ou consultório	74 (91,3)	66 (80,4)	0,06
Clínica de diálise	53 (65,4)	59 (71,9)	0,30
Busca de conhecimento, N (%)			
Artigos científicos	77 (95)	75 (91,4)	0,36
Livros	38 (46,9)	44 (53,6)	0,39
Cursos e congressos	63 (77,7)	61 (74,4)	0,61
Redes sociais	25 (30,8)	42 (51,2)	0,008
Aulas virtuais	60 (74,0)	56 (68,3)	0,41

Abreviações – N: número; RM: residência médica.

Nota – *1 participante atua fora do Brasil.

A maioria dos profissionais atua predominantemente na esfera assistencial, em hospitais e ambulatórios. Observa-se uma tendência de maior atuação em ambulatórios entre os profissionais que concluíram a residência antes de 2012, em comparação com aqueles formados a partir desse ano (91,3% vs. 80,4%; p = 0,06).

De modo geral, não houve diferenças significativas entre os grupos em relação às principais fontes de atualização profissional, com predominância da leitura de artigos científicos, seguida pela participação em cursos e congressos. A busca por conhecimento por meio de redes sociais foi a menos mencionada pelos participantes; contudo, aqueles que concluíram a RM após 2012 relataram utilizá-las em maior proporção (51,2% vs. 30,8%; p = 0,008).

Os grupos também diferiram em relação à percepção de algumas competências clínicas adquiridas ao final da formação (Tabela 3). Todos os participantes que concluíram o processo formativo após 2012 afirmaram sentir-se aptos a realizar o tratamento conservador da DRC, em contraste com 95% daqueles que finalizaram antes desse período (p = 0,04). A percepção de aprendizado da competência “reconhecer e abordar terminalidade” foi significativamente mais frequente entre os egressos após 2012 (72 vs. 35,8%; p < 0,001). Por outro lado, os profissionais formados antes de 2012 apresentaram maiores proporções de autopercepção de competência para realizar fundoscopia (30,9% vs. 18,3%; p = 0,03) e para implantar cateter de DP (35,8% vs. 14,6%) (Tabela 3).

**Tabela 3:** Percepção do aprendizado (aptidão) e da relevância atual (utilidade) de competências desenvolvidas durante a residência médica em nefrologia, segundo o tempo de conclusão da formação

Variável	Total (n = 163)	Conclusão até 2012 (N = 81)	Conclusão após 2012 (N = 82)	p
Realizar consulta ambulatorial				
Apto, N (%)	128 (78,5)	63 (77,8)	65 (79,3)	0,89
Útil, N (%)	153 (93,8)	76 (93,8)	77 (93,9)	0,78
Diagnosticar e tratar GN				
Apto, N (%)	119 (73,0)	59 (72,8)	60 (73,2)	0,77
Útil, N (%)	152 (93,2)	75 (92,6)	77 (93,9)	0,56
Realizar tratamento conservador				
Apto, N (%)	159 (97,5)	77 (95,1)	82 (100)	0,04
Útil, N (%)	162 (100)	81 (100)	81 (100)	
Prescrever e acompanhar HD				
Apto, N (%)	158 (96,9)	77 (95,1)	81 (98,8)	0,35
Útil, N (%)	156 (95,7)	74 (91,4)	82 (100)	0,02
Prescrever e acompanhar DP				
Apto, N (%)	119 (73,0)	60 (74,1)	59 (72,0)	0,93
Útil, N (%)	121 (74,2)	63 (77,8)	58 (70,7)	0,37
Acompanhar transplantado renal				
Apto, N (%)	92 (56,4)	52 (64,2)	40 (48,8)	0,14
Útil, N (%)	100 (61,7)	52* (65,0)	48* (59,3)	0,75
Diagnosticar e tratar IRA				
Apto, N (%)	152 (93,8)	73 (90,1)	79* (97,5)	0,13
Útil, N (%)	150 (92,6)	71 (87,7)	79* (97,5)	0,05
Reconhecer e abordar terminalidade				
Apto, N (%)	88 (53,9)	29 (35,8)	59 (72,0)	<0,001
Útil, N (%)	152 (93,2)	74 (91,4)	78 (95,1)	0,62
Realizar exame de fundo de olho				
Apto, N (%)	40 (24,5)	25 (30,9)	15 (18,3)	0,02
Útil, N (%)	86 (53,1)	44* (55,0)	42 (51,2)	0,78
Realizar biópsia renal				
Apto, N (%)	82 (50,3)	47 (58,0)	35 (42,7)	0,14
Útil, N (%)	75 (46,0)	41 (50,6)	34 (41,5)	0,41
Implantar cateter provisório para HD				
Apto, N (%)	150 (92,6)	73 (90,1)	77* (95,1)	0,36
Útil, N (%)	140 (85,9)	70 (86,4)	70 (85,4)	0,79
Implantar cateter definitivo para HD				
Apto, N (%)	33 (20,2)	73 (90,1)	77* (95,1)	0,59
Útil, N (%)	51 (31,2)	70 (86,4)	70 (85,4)	0,68
Implantar cateter para DP				
Apto – N (%)	41 (25,1)	16 (19,8)	17 (20,7)	<0,001
Útil – N (%)	50 (30,6)	24 (29,6)	27 (32,9)	0,23
Realizar USG à beira leito				
Apto – N (%)	55 (33,7)	29 (35,8)	12 (14,6)	<0,001
Útil – N (%)	123 (75,4)	29 (35,8)	21 (25,6)	0,001
Realizar trabalho colaborativo				
Apto, N (%)	141 (86,5)	03 (3,7)	52 (63,4)	0,09
Útil, N (%)	156 (95,7)	51 (63,0)	72 (87,8)	0,43
Liderar equipes				
Apto, N (%)	95 (58,2)	66 (81,5)	75 (91,5)	0,22
Útil, N (%)	142 (87,1)	78 (96,3)	78 (95,1)	0,80
Gerenciar serviços de nefrologia				
Apto, N (%)	19 (11,6)	42 (51,9)	53 (64,6)	0,14
Útil, N (%)	96 (59,2)	71 (87,7)	71 (86,6)	0,10
Gerenciar a carreira				
Apto, N (%)	61 (37,4)	06 (7,4)	13 (15,9)	0,22
Útil, N (%)	112 (69,1)	54* (67,5)	42 (51,2)	0,15
Realizar atividades docentes				
Apto, N (%)	44 (26,9)	25 (30,9)	36 (43,9)	0,05
Útil, N (%)	87 (53,3)	57* (71,3)	55 (67,1)	0,008
Realizar pesquisa clínica				
Apto, N (%)	23 (14,1)	08 (9,9)	15 (18,3)	0,20
Útil, N (%)	136 (83,9)	65* (81,3)	71 (86,6)	0,50

Abreviações – N: número; RM: residência médica; GN: glomerulonefrite; HD: hemodiálise; DP: diálise peritoneal; IRA: injuria renal aguda; USG: ultrassonografia.

Nota – *1 participante não respondeu.

Outra diferença observada entre os grupos refere-se à percepção da utilidade de determinados componentes formativos. Aqueles que concluíram a RM antes de 2012 atribuíram menor utilidade ao acompanhamento de pacientes em HD (91,4% vs. 100%; p = 0,025) e à realização de USG (63% vs. 87,8%; p = 0,001), ao passo que consideraram mais útil a participação em atividades docentes (65,4% vs. 41,5%; p = 0,008), em comparação com os que finalizaram após 2012.

## Discussão

Este estudo, que incluiu 3,32% dos nefrologistas brasileiros, oferece uma análise da percepção desses profissionais sobre a RM em nefrologia. Trata-se de uma amostra composta por profissionais mais jovens do que os descritos no estudo “Demografia Médica no Brasil 2023”^13^, o que possivelmente se explica pelo uso de método on-line para coleta das informações e pelo perfil dos pesquisadores, que possuem maior contato com nefrologistas com menor tempo de atuação.

A amostra abrangeu profissionais oriundos de programas de RM de diversas regiões do Brasil, sendo que a maioria dos participantes realizou a residência no estado de São Paulo, local com o maior número de programas de RM^1^. Trata-se de uma amostra de conveniência, com representação proporcionalmente menor das regiões Sul e Norte, o que dificulta a extrapolação dos dados para essas localidades.

Os resultados mostram que, embora a RM prepare adequadamente os nefrologistas para as atividades clínicas essenciais, ainda há lacunas significativas em competências específicas, especialmente na abordagem da terminalidade e na área intervencionista.

Os cuidados paliativos são um tópico historicamente negligenciado pela educação médica e, consequentemente, pelos programas de RM em nefrologia. O contraste entre a falta de preparo e a percepção da necessidade de seu aprendizado também foi relatado em diversos estudos internacionais^11,14–16^. No Brasil, apenas em 1980 iniciou-se o uso dos cuidados paliativos como filosofia de trabalho e, somente em 2023, sua inclusão foi oficializada nas diretrizes curriculares nacionais dos cursos de Medicina^17^. Até os dias atuais, muitos serviços de nefrologia, inclusive alguns que oferecem programas de RM, ainda não possuem diretrizes bem estabelecidas acerca do tema^18^.

Os dados do presente estudo sugerem que o aprendizado em cuidados paliativos melhorou na última década e que sua inclusão na matriz de competências da SBN^5^ contribuiu para direcionar o olhar dos programas de RM para essa questão. Entretanto, faz-se necessário conferir maior protagonismo ao tema, por meio de sua inclusão no projeto educacional e no ementário dos cursos teóricos, bem como por meio de treinamentos práticos específicos, inclusive com a possibilidade de vivências interdisciplinares em serviços especializados de cuidados paliativos.

O aprendizado das demais aptidões clínicas analisadas foi semelhante entre os participantes que realizaram a RM antes e após 2012, com exceção da percepção sobre o tratamento conservador da DRC, que foi maior entre aqueles que concluíram a RM mais recentemente.

Em relação à utilidade das diferentes aptidões clínicas, observou-se uma menor percepção da utilidade da competência “prescrever e acompanhar HD” entre os participantes que concluíram a RM há mais tempo, em comparação aos egressos mais recentes. Esse achado pode estar relacionado a diferenças no perfil de atuação profissional entre os grupos: os nefrologistas formados até 2012 relataram maior atuação em ambulatórios ou consultórios, enquanto aqueles formados a partir de 2012 referiram maior presença em hospitais e clínicas de HD.

Dentre os procedimentos avaliados, a realização de fundoscopia, o implante de cateter definitivo para HD, bem como o implante de cateter peritoneal, e o uso da USG à beira do leito foram aqueles em que os nefrologistas se consideraram menos aptos a realizar ao final da RM.

O exame de FO, segundo alguns autores, deveria tornar-se parte integrante do exame físico geral^19,20^. No entanto, seu ensino na graduação em Medicina e sua prática regular por médicos não oftalmologista são negligenciados, possivelmente devido às dificuldades técnicas que esses profissionais encontram para realizar e interpretar o exame^19,20^. Provavelmente, essa habilidade tem sido historicamente menos ensinada na RM por ter saído da rotina da prática nefrológica ao longo dos anos.

Recentemente, tem-se debatido se procedimentos anteriormente considerados indispensáveis à prática do nefrologista, como a inserção de cateter para HD e a realização de biópsia renal, devem continuar sendo realizados por esse especialista^21–24^. No presente estudo, observou-se que, na maioria dos centros avaliados, o nefrologista realiza ambos os procedimentos. No entanto, apenas a passagem de cateter provisório para HD foi considerada útil pela maioria dos participantes (apenas 46% consideram a realização de biópsia renal uma habilidade útil na prática atual).

Diversos fatores podem contribuir para esses achados, como a sobrecarga de trabalho, a má remuneração, dificuldades logísticas, falta de oportunidades para a prática e ausência de treinamento específico^23,24^. O aprendizado segue a lógica do serviço em que o residente está inserido, e muitos deles sequer oferecem a oportunidade de treinamento específico, uma vez que os diferentes procedimentos são realizados por profissionais de outras especialidades, como cirurgiões e radiologistas^24^.

No tocante ao implante de cateter peritoneal, o cenário se revela ainda mais desafiador: apenas 14,2% dos egressos a partir de 2012 afirmaram ter desenvolvido essa competência durante a RM, menos da metade do percentual observado no outro grupo (35,8%). É possível que a progressiva redução da utilização da modalidade de DP como método terapêutico no país^25^ justifique parte dos dados, considerando que alguns serviços de RM sequer oferecem a modalidade.

Esses resultados suscitam uma importante reflexão sobre a realização de procedimentos pelo nefrologista. Já foi demonstrado que o envolvimento de diferentes profissionais fragmenta o cuidado ao paciente, atrasa o início e aumenta o custo do tratamento^26,27^. Além disso, um estudo anterior com 239 nefrologistas em atuação no Brasil mostrou que 90% deles teriam interesse em receber maior treinamento em nefrologia intervencionista^28^. Contudo, não há regulamentação acerca do número mínimo de procedimentos necessários para a certificação do médico nefrologista nessas técnicas, além de ser necessário dispor de um serviço especializado, com a presença de médicos de outras áreas, como vascular, cirurgião geral e urologista, para o manejo de possíveis complicações^23,24,29^.

Diante desse cenário, torna-se necessário reavaliar as práticas formativas. Caso esses procedimentos continuem sendo considerados competências obrigatórias durante a RM em nefrologia, os programas devem estruturar estratégias eficazes de ensino-aprendizagem. Uma alternativa seria a oferta de estágios optativos em centros especializados, direcionados aos residentes com interesse específico em nefrologia intervencionista, os quais poderiam atuar como multiplicadores em seus respectivos serviços.

A realização de USG tem sido recomendada para guiar a passagem de cateter de HD, a realização de biópsia renal e, mais recentemente, com a introdução da USG *point-of-care* (POCUS), como ferramenta diagnóstica^5,30^. Há relatos de que o uso da USG melhora a segurança do paciente, aumenta a satisfação com o cuidado e reduz os custos do tratamento^11,30^. Contudo, para sua implementação concreta, faz-se necessário dispor de equipamentos de USG e de preceptores habilitados para o ensino dessa prática^31^. Este estudo mostra que a formação em USG à beira do leito é considerada de utilidade prática pelos nefrologistas, e seu aprendizado vem aumentando, sendo mais frequente entre aqueles que concluíram a RM mais recentemente.

O panorama descrito neste estudo reflete uma tendência observada internacionalmente. De maneira semelhante, uma pesquisa conduzida com residentes de nefrologia na Itália destacou a necessidade de aprimorar a formação em USG, cuidados paliativos, nutrição clínica e acesso vascular^32^.

Além do cuidado ao paciente, a RM deve possibilitar que o nefrologista expanda seu currículo e desenvolva competências em gestão, liderança, pesquisa e ensino. Neste estudo, a maioria dos nefrologistas considerou que a RM não lhes proporcionou o desenvolvimento dessas competências específicas, apesar de as considerarem úteis para sua prática atual. Estudos internacionais corroboram esses achados. Uma pesquisa realizada na Nova Zelândia mostrou preparação adequada para a maioria das áreas clínicas, mas revelou fragilidades nos domínios de pesquisa e gestão^12^. Já o estudo conduzido por Berns em 2010, revelou que 70% dos participantes relataram ter recebido nenhum ou pouco treinamento em gestão de serviços de nefrologia^10^.

Adquirir uma visão geral sobre gestão é essencial ao nefrologista, que, de maneira geral, realiza seu trabalho de forma interdisciplinar, muitas vezes liderando equipes, no exercício ou não de funções administrativas^4,5,11^. Para desenvolver o aprendizado nessa área, o residente deveria receber treinamento formal em liderança e gestão. Isso poderia incluir a participação em atividades administrativas reais, como reuniões de gestão, planejamento de equipe e organização de fluxos assistenciais, além de módulos teórico-práticos sobre temas como gestão de tempo, comunicação não violenta, feedback, gestão de pessoas, gestão financeira, administração, faturamento, contratos e regulação em saúde.

Os resultados também destacam o baixo preparo durante a RM para a realização de atividades docentes e de pesquisa, o que pode impactar negativamente a formação de novos nefrologistas. Aumentar a participação do nefrologista em atividades acadêmicas é uma possível estratégia para reverter a queda do interesse pela especialidade^7,33^. Para isso, é fundamental que os residentes aprendam sobre os princípios da aprendizagem de adultos, planejamento educacional, estratégias educacionais e avaliação, sendo inseridos de forma mais ativa em atividades acadêmicas, como aulas, seminários e preceptorias supervisionadas. Além disso, devem ser incentivados a participar de projetos de pesquisa desenvolvidos nos serviços e ter acesso a uma formação estruturada em metodologia científica, abordando desde o desenho de estudos até a análise crítica da literatura, redação de documentos científicos e produção acadêmica.

A qualidade da formação do nefrologista está intrinsecamente ligada à existência de programas de RM com planejamento educacional estruturado, que sejam abrangentes, atualizados e responsivos às transformações da prática médica. A avaliação sistemática e contínua desses programas é fundamental para garantir que a formação acompanhe as exigências da sociedade, do sistema de saúde e da própria especialidade^12^.

### Limitações

O presente estudo possui limitações. O questionário foi desenvolvido pelos autores, seu conteúdo foi revisado e reformulado a partir de sugestões de alguns nefrologistas, mas não foi validado antes de sua aplicação. Apesar de ter sido baseado na matriz de competências da SBN, o instrumento não compreendeu todas as competências descritas no documento.

Além disso, o questionário foi distribuído por meio dos contatos pessoais dos autores, o que pode ter selecionado uma população mais jovem e conectada. Apesar dos esforços empreendidos, a pesquisa contou com a participação de um número limitado de nefrologistas, o que pode ter introduzido viés de seleção. A maioria dos participantes são da região Sudeste, e houve baixa representatividade das regiões Norte e Sul do Brasil, o que pode limitar a generalização dos resultados para outras regiões do país.

Os resultados baseiam-se na percepção subjetiva dos participantes sobre a aquisição e a utilidade das competências desenvolvidas durante a RM, o que pode estar sujeito a viés de memória. Não foram realizadas avaliações objetivas para mensurar o real domínio dessas competências, nem sua aplicabilidade no sistema de saúde ou na comunidade.

Por fim, o estudo incluiu nefrologistas que realizaram sua formação em diferentes períodos. Como os programas de RM passaram por evoluções ao longo do tempo, é possível que haja discrepâncias na percepção do aprendizado entre diferentes gerações de especialistas.

### Implicações Clínicas

Os achados deste estudo ressaltam a necessidade de aprimoramento contínuo da RM em nefrologia, com ênfase na melhoria do ensino de cuidados paliativos, USG à beira do leito, gestão de serviços de saúde e educação médica. O processo de ensino-aprendizagem, durante a RM, de procedimentos como biópsia renal, implante de cateter definitivo para HD e cateter para DP deve ser reavaliado.

A implementação de avaliações sistemáticas dos egressos dos programas de RM pode fornecer subsídios valiosos para ajustes curriculares mais eficazes, alinhando a formação médica às demandas da sociedade e do sistema de saúde. Além disso, essas avaliações podem orientar o desenvolvimento de programas de educação continuada, assegurando que os nefrologistas estejam preparados para os desafios contemporâneos da especialidade, promovendo um atendimento mais eficiente.

## Conclusão

A RM em nefrologia no Brasil cumpre adequadamente sua função formativa; porém, ainda apresenta lacunas em áreas como cuidados paliativos, procedimentos intervencionistas, ensino, pesquisa e gestão, que precisam ser abordadas para uma formação mais completa do nefrologista.

Espera-se que esta pesquisa contribua para o aprimoramento dos programas de RM em nefrologia, por meio da avaliação sistemática dos egressos e da implementação de programas de educação continuada, permitindo ajustes curriculares que atendam às necessidades da sociedade e da própria especialidade.

## Data Availability

Os conjuntos de dados gerados e/ou analisados durante o presente estudo estão disponíveis mediante solicitação razoável ao autor correspondente.

## Material Suplementar

O seguinte material on-line está disponível para o presente artigo:

Anexo 1 Questionário.

Anexo 2 Termo de Consentimento Livre e Esclarecido.
